# Pilot randomized controlled trial of a Mediterranean diet or diet supplemented with fish oil, walnuts, and grape juice in overweight or obese US adults

**DOI:** 10.1186/s40795-018-0234-y

**Published:** 2018-05-31

**Authors:** Lindsay M. Jaacks, Salman Sher, Christine De Staercke, Markus Porkert, Wayne R. Alexander, Dean P. Jones, Viola Vaccarino, Thomas R. Ziegler, Arshed A. Quyyumi

**Affiliations:** 1 Department of Global Health and Population, Harvard T.H. Chan School of Public Health, 665 Huntington Ave, Building 1, Room 1211, Boston, MA 02115 USA; 2 Division of Cardiology, Department of Medicine, Emory University School of Medicine, Atlanta, GA 30307 USA; 3 Hemostasis Laboratory Branch, Division of Blood Disorders, National Center on Birth Defects and Developmental Disabilities, Centers for Disease Control and Prevention, Atlanta, GA 30329 USA; 4 Gwinnett Medical Center, Lawrenceville, GA 30046 USA; 5 Division of Pulmonary, Allergy, Critical Care and Sleep Medicine, Department of Medicine, Emory University School of Medicine, Atlanta, GA 30307 USA; 6 Department of Epidemiology, Rollins School of Public Health, Emory University, Atlanta, GA 30322 USA; 7 Division of Endocrinology, Metabolism and Lipids, Department of Medicine, Emory University School of Medicine, Atlanta, GA 30307 USA

**Keywords:** Mediterranean diet, Omega-3 fatty acids, Hyperlipidemia, Endothelial function, Oxidative stress

## Abstract

**Background:**

The 2015–2020 Dietary Guidelines for Americans recommend a Mediterranean-type diet as one of three healthful eating patterns. However, only one previous trial has evaluated the effects of a Mediterranean diet intervention in a US sample population.

**Methods:**

To address this gap, we conducted a pilot, non-blinded, 8-week randomized controlled trial on the comparative efficacy of consumption of a Mediterranean diet or a diet supplemented with fish oil, walnuts, and grape juice versus controls. Participants (overweight or obese US adults; 73% female and mean age 51 years) were randomly assigned to one of three groups: (1) Mediterranean diet; (2) habitual high-fat American-type diet supplemented with fish oil, walnuts, and grape juice; or (3) habitual high-fat American-type diet (controls). Intent-to-treat analysis of within-subject differences (Student’s paired t-test or Wilcoxon sign ranks test) and between-subject differences (mixed-effects models with a group-by-time interaction term, adjusted for baseline health outcome) was conducted.

**Results:**

Participants in the Mediterranean diet arm (*n* = 11) had significantly greater weight loss despite no significant change in total caloric intake, and lower plasma cystine, indicative of decreased oxidative stress, compared to controls (*n* = 9) at both 4 and 8 weeks. Compared to controls, they also had significantly lower total cholesterol and low-density lipoprotein cholesterol levels at 4 weeks. Participants in the supplement arm (*n* = 10) had significantly lower adiponectin levels compared to controls at 4 weeks. No significant improvements in endothelial function or inflammatory biomarkers were observed in either intervention group compared to controls.

**Conclusion:**

These results suggest that adopting a dietary pattern reflecting a Mediterranean diet improves weight and cardio-metabolic health among overweight or obese US adults, and may be more beneficial than supplementing habitual American diets with fish oil, walnuts, and grape juice.

**Trial registration:**

ClinicalTrials.gov NCT00166088. Registered 14 September 2005.

## Background

A Mediterranean diet supplemented with either olive oil or nuts is the only dietary intervention that has been shown to prevent major cardiovascular events in a randomized controlled trial [1]. Consistent with this, several smaller trials of the Mediterranean diet conducted in Italy, Greece, Spain, France, and Finland, have variously reported protective effects against cardiovascular disease (CVD) risk factors including adiposity, hypercholesterolemia, hyperglycemia, insulin resistance, endothelial dysfunction, oxidative stress, and/or inflammation [2–5].

Despite new recommendations in the 2015–2020 Dietary Guidelines for Americans suggesting that a Mediterranean-type diet is healthful [6], only one trial, to our knowledge, has evaluated the effects of a Mediterranean diet intervention in a US population [7]. In that study of non-obese adult women in Michigan, guidance on Mediterranean diet intake using a dietary component exchange list was coupled with serial counseling by a dietitian for adherence based on food records at baseline, 3 months, and 6 months [7]. Compared to the control group following a habitual diet, no changes in blood lipids, except for an increase in plasma monounsaturated fatty acids, were noted in the Mediterranean diet group [7].

It is unclear from previous studies whether the Mediterranean diet as a whole is necessary to see improvements in health, or if habitual diets can be supplemented with key components of the Mediterranean diet with similar benefit. It has been hypothesized that much of the benefit of the Mediterranean diet stems from omega-3 polyunsaturated fatty acids, including eicosapentaenoic acid (EPA) and docosahexaenoic acid (DHA) found in fish, and α-linolenic acid found in nuts [8, 9]. Moderate alcohol consumption, especially the phytochemicals found in wine, is also thought to provide cardio-metabolic benefit [10, 11].

The rationale for this study is therefore two-fold: (1) to establish whether or not a Mediterranean-type diet is protective against CVD risk factors in a US population and (2) whether or not supplementing a habitual high-fat American-type diet with several key components of the Mediterranean diet can produce similar reductions in CVD risk factors. Here we report results of a pilot, non-blinded, 8-week randomized controlled trial to test whether a Mediterranean diet or a habitual high-fat American-type diet supplemented with several key components of the Mediterranean diet reduces CVD risk factors compared to a habitual high-fat American-type control diet among overweight or obese adults.

## Methods

### Sample population

Participants were recruited from Emory Healthcare outpatient clinics and by advertisements. Assessments were conducted by trained study staff at the Emory University Hospital Clinical Research Center (CRC), formerly the General Clinical Research Center, of the Atlanta Clinical and Translational Science Institute. The study was approved by the Emory University Institutional Review Board and all participants provided written informed consent.

The inclusion criteria were as follows: aged 40–65 years, body mass index (BMI) ≥28 kg/m^2^ and < 35 kg/m^2^, stable body weight in the past 6 months (defined as weight change < 2 kg), currently a non-smoker, currently consuming a stable habitual diet as assessed by interview with the research nutritionist, not consuming anti-oxidants or vitamin-mineral preparations in the past 4 weeks, and 24-h diet recall showing saturated and trans fat intake of > 10% of total daily calories and total cholesterol intake of > 300 mg/day. Participants meeting any of the following criteria were excluded: known history of cardiovascular diseases including coronary artery disease, valvular heart disease, arrhythmias or cardiomyopathies; renal or liver disease defined as creatinine > 2.0 mg/dL and liver function tests > 3-times upper limit of normal; history of diabetes or fasting plasma glucose (FPG) > 126 mg/dL; history of cancer other than skin cancer; systolic blood pressure > 180 mmHg and diastolic blood pressure > 110 mmHg; hematocrit < 30%; and other known acute or chronic illness, including psychiatric disorders, excessive chronic alcohol consumption (> 2 alcoholic beverages/day), statin or other hypolipidemic therapy, and pregnant or lactating females. No imaging was performed for exclusion of fatty liver disease.

Following a screening visit, participants were randomly assigned to one of three groups: (1) Mediterranean diet; (2) habitual high-fat American-type diet supplemented with fish oil, walnuts, and grape juice daily; or 3) habitual high-fat American-type diet (controls). Study measurements were made at a baseline visit and again at 4 and 8 weeks after the baseline visit.

### Mediterranean diet intervention

Participants randomized to the Mediterranean diet arm received three meals with beverages and two snacks per day as a prototypical Mediterranean diet prepared by the CRC metabolic kitchen for four consecutive weeks, coupled with verbal and written dietary instruction. During the first four-week period, meals and snacks were picked up by participants at the CRC metabolic kitchen every three to four days and any issues with the meal plan were discussed with the research nutritionist. The seven-day menu from the first week was rotated on a day-to-day basis during the subsequent three weeks to avoid monotony. Verbal and written dietary instruction on the advantages and composition of the Mediterranean diet were given by the CRC research nutritionist during the first four-week period. During the second four-week period, participants received intensive verbal and detailed written dietary instruction and reinforcement to ensure consumption of a prototypical Mediterranean diet using their own home-cooked meals. The CRC research nutritionist formally discussed and reinforced dietary principles with the participant weekly, either in person (baseline, weeks 2, 4, and 6) or via telephone (weeks 1, 3, 5, and 7). Appropriate adjustments were made, as needed, to individual food items to ensure high compliance.

Meals and food plans were designed using ProNutra™ (Viocare, Inc., Princeton, NJ) to provide daily energy for weight maintenance as determined by the Harris-Benedict equation [12]. Protein intake was provided at the Recommended Dietary Allowance level of 0.8 g/kg/day; saturated and *trans* fats at < 7% of total energy intake; and cholesterol at < 200 mg/day. Using ProNutra™, meals for individual participants were based on the diet scoring for Mediterranean diet adherence [13]. Meals included an abundance of plant food (fruits, vegetables, whole grains, nuts, and legumes); olive oil as the primary source of fat; fish, poultry, and eggs in moderate to low amounts; low consumption of red meats, saturated fats, and sweets; and consumption of either one to two ounce drinks per day of wine (in those who habitually consumed wine) or grape juice (in those who did not habitually consume alcohol).

### Diet supplementation intervention

Participants assigned to their habitual high-fat American-type diet supplemented with key components of the Mediterranean diet were given an eight-week supply of specific dietary supplements by the CRC nutritionist and advised to consume them daily, in addition to their usual diet. They were also advised to decrease caloric intake from usual dietary constituents (e.g., not the dietary supplements) if they observed body weight gain of more than 0.5 kg during any given week. The supplements included: (1) two 1-g pills of Omacor® fish oil supplements (1.8 g of EPA/DHA) per day, (2) 1/3 cup shelled walnuts per day, and (3) 16 ounces (about 475 mL) Welch’s® 100% Concord grape juice per day.

### Control group

These participants did not alter their diet, take supplements, alter usual activity, and were not given dietary advice.

### Dietary intake assessment

Participants were asked to complete 3-day food records at baseline and again at 4 and 8 weeks after the baseline visit. Nutrient composition was determined using ProNutra™ (Viocare, Inc., Princeton, NJ). The 3-day average nutrient intake at each time point was used in the final analysis.

### Outcome assessment

Anthropometric measurements (weight and waist circumference) were assessed using standardized procedures by trained CRC staff. Blood lipids (total cholesterol, triglycerides, high-density lipoprotein [HDL] cholesterol, and low-density lipoprotein [LDL] cholesterol) were measured using a CX7 chemistry analyzer (Beckman Diagnostics, Fullerton, CA) by technicians at the Emory Healthcare Medical Laboratory. Inflammatory biomarkers and adipokines included IL-6, IL-8, C-reactive protein (CRP), and adiponectin. IL-6 and IL-8 were measured with a Fluorokine MAP MultiAnalyte Profiling Human Base Kit (R&D Systems, Inc., Minneapolis, MN) on a Luminex – 200 platform. IL-6 values that were below the limit of detection were assigned a value of 0.01 pg/mL. CRP was measured using the Dade-Behring Nephelometry System (BNII). One participant had a CRP level less than the limit of detection (LOD) and was assigned a value of LOD/sqrt(2). Adiponectin was measured with the Quantikine Human Adiponectin Immunoassay solid-phase ELISA (R&D Systems, Inc., Minneapolis, MN). Markers of hyperglycemia, including FPG and insulin were measured using a CX7 chemistry analyzer (Beckman Diagnostics, Fullerton, CA) by technicians at the Emory Healthcare Medical Laboratory.

Brachial artery reactivity was used to test flow-mediated vasodilation (FMD) as a measure of endothelial function and more specifically of nitric oxide bioavailability. This outcome was chosen because endothelial dysfunction integrates risk factor-mediated injury to the endothelial cells. It is an early marker of risk for development of atherosclerosis and its adverse outcomes and thus provides a sensitive, reproducible and non-invasive tool for investigation of the effects of short-term dietary intervention (e.g., an excellent experimental tool) [14, 15]. All measurements were performed in a temperature-controlled laboratory after an overnight fast. The brachial artery of the non-dominant arm was imaged using a high-resolution 10 MHz linear array ACUSON™ ultrasound transducer (Siemens Medical Solutions USA, Inc., Malvern, PA) at baseline and continually for 120 s after producing 5-min ischemia of the hand. Arterial diameter was measured as the distance from the leading edge of the intima-lumen interface of the near wall to the leading edge of the lumen-intima interface of the far wall by an investigator blinded to the treatment. FMD was calculated as the percent increase in brachial artery vasodilator response: (post-hyperemia diameter-baseline diameter)/baseline diameter × 100.

Circulating pro-angiogenic cell activity (CFU-As) were measured using a colony forming assay from circulating mononuclear cells as described previously [16, 17]. Briefly, mononuclear cells were isolated by density-gradient centrifugation from a 20 ml sample of venous blood using CPT tubes (Becton, Dickinson and Company, Franklin Lakes, NJ), and washed two times with PBS. The cells were suspended in growth medium (DMEM supplemented with 20% fetal bovine serum and 6.5% endothelial cell growth supplement), and plated on human fibronectin-coated cell culture dishes. To eliminate mature circulating endothelial cells, cells adherent after 24 h were discarded and nonadherent cells were re-plated onto new fibronectin-coated plates at 1 million cells/well. Growth medium was changed every 2 days. After 7 days, CFU-As were counted manually and recorded by an observer who was blinded to the clinical data. Colonies were identified as central clusters of rounded cells with multiple flat cells emanating from the central clusters.

Biomarkers of oxidative stress included plasma cysteine (reduced form), cystine (oxidized form), and glutathione (reduced form), and were measured using high-performance liquid chromatography with fluorescence detection as described previously [18].

### Statistical analysis

Values presented are mean with standard deviation given in parentheses. Normality was tested using the Shapiro-Wilk test statistic and visually assessed using Q-Q plots. Differences in demographic, clinical, and dietary factors between arms at baseline were assessed using Fisher’s exact test for categorical variables and analysis of variance (ANOVA) for continuous variables. Within-subject differences in the health outcomes were analyzed using Student’s paired t-test for normally distributed variables and Wilcoxon sign ranks test for non-normally distributed variables [19, 20]. Mixed-effects models were used to analyze between-subject differences in the health outcomes [21]. Models included a group-by-time interaction term. Baseline health outcome was adjusted in all models. Statistical significance was considered for *P* < 0.05. All analyses were conducted using SAS version 9.4 (SAS Institute, Cary, NC).

## Results

48 individuals were screened for inclusion. Of these, 11 (23%) were ineligible (*n* = 9 for BMI < 28 kg/m^2^ or ≥ 35 kg/m^2^ and *n* = 2 for cancer history) and 37 were randomly assigned to control (*n* = 11), Mediterranean diet (n = 11), or diet supplements (*n* = 15). Of these, *n* = 7 (19%; n = 2 control and *n* = 5 supplement) were non-compliant with follow-up visits and excluded. The final sample was therefore 9 randomized to control, 11 to Mediterranean diet, and 10 to supplements. Participants were 51.4 (6.6) years old and 73.3% female.

### Change in dietary intake

Participants in the Mediterranean diet arm exhibited a significant decrease in their saturated fat and cholesterol intake, and a significant increase in monounsaturated, omega-3, and omega-6 fatty acid intakes compared to controls at 4 weeks (Table 1). These differences were attenuated and no longer statistically significant by 8 weeks. Participants in the supplement arm had significantly higher total fat, monounsaturated, omega-3, and omega-6 fatty acid intakes compared to controls at both 4 and 8 weeks.

**Table 1:** Characteristics of participants at baseline and after 4 and 8 weeks of the dietary intervention^a,b^

	Control *n* = 9			Mediterranean Diet *n* = 11			Supplements (Fish Oil, Walnuts, and Grape Juice) *n* = 10		
	Baseline	4 weeks	8 weeks	Baseline	4 weeks	8 weeks	Baseline	4 weeks	8 weeks
Dietary variables									
Energy, kcal	2159 ± 411	2047 ± 436	1964 ± 680	2346 ± 742	2368 ± 486	2192 ± 1110	1647 ± 494	2163 ± 775*	2266 ± 321*
Tot fat, %kcal	40.3 ± 4.8	40.8 ± 5.7	32.1 ± 5.6	40.4 ± 5.3	35.1 ± 2.6	31.8 ± 10.2	34.4 ± 5.8	43.4 ± 5.8*	43.3 ± 4.3*
Sat fat, %kcal	12.8 ± 2.9	14.2 ± 3.5	9.7 ± 2.2	12.5 ± 4.6	5.8 ± 1.5*	7.6 ± 2.7	11.0 ± 3.2	8.6 ± 3.2	8.7 ± 1.5
Carb, %kcal	44.3 ± 9.0	44.9 ± 7.3	50.5 ± 6.1	47.4 ± 6.2	52.0 ± 3.9	51.0 ± 7.6	45.0 ± 15.1	41.0 ± 9.0	42.4 ± 4.5
Protein, %kcal	17.1 ± 3.2	15.5 ± 3.4	18.1 ± 3.2	14.0 ± 3.9	15.9 ± 1.5	16.9 ± 3.1	19.4 ± 6.1	16.9 ± 3.4	15.0 ± 1.7*
Fiber, g	18.1 ± 3.8	19.2 ± 9.5	21.2 ± 9.99	19.7 ± 9.0	29.6 ± 10.1	28.7 ± 10.0	20.6 ± 12.7	22.3 ± 9.8	19.2 ± 6.6
Chol, mg	292 ± 73	306 ± 128	282 ± 136	362 ± 216	223 ± 35.4*	334 ± 459	269 ± 120	301 ± 63.6	277 ± 47.4
MUFA, g	23.0 ± 6.8	17.3 ± 6.0	18.1 ± 10.6	16.9 ± 13.6	41.3 ± 10.0*	29.3 ± 20.1	14.4 ± 6.8	24.1 ± 9.7*	27.2 ± 4.6*
Omega-3, g	0.81 ± 0.74	0.58 ± 0.22	0.91 ± 0.69	0.81 ± 0.96	2.25 ± 0.76*	1.97 ± 2.32	0.47 ± 0.28	5.92 ± 1.39*	5.62 ± 1.31*
Omega-6, g	7.62 ± 3.65	4.54 ± 1.66	6.00 ± 3.13	7.09 ± 7.76	21.5 ± 6.89*	12.6 ± 12.5	4.25 ± 2.87	26.6 ± 10.0*	25.5 ± 2.61*
Clinical variables									
Weight, kg	92.7 ± 7.2	96.2 ± 12.4	96.6 ± 11.1	93.4 ± 12.8	91.7 ± 12.5*	90.5 ± 13.0*	98.6 ± 16.8	99.5 ± 16.9	99.8 ± 16.8
WC, cm	105.7 ± 9.2	112.0 ± 12.3	107.4 ± 10.5	107.3 ± 6.7	107.9 ± 6.5	104.5 ± 5.9	114.1 ± 11.4	114.7 ± 11.5	112.8 ± 17.0
TC, mg/dl	189.6 ± 42.6	190.3 ± 30.9	179.3 ± 32.3	187.4 ± 38.4	163.3 ± 36.0*	164.2 ± 36.2	185.4 ± 19.9	178.3 ± 21.3	180.8 ± 19.4
TG, mg/dl	80.4 ± 37.3	84.5 ± 34.3	97.9 ± 51.7	141.9 ± 110.9	140.5 ± 100.6	109.3 ± 50.3	82.1 ± 26.9	66.0 ± 20.5	59.9 ± 19.7
HDL, mg/dl	46.2 ± 12.8	43.5 ± 11.9	40.9 ± 12.1	43.8 ± 14.1	40.2 ± 10.7	45.1 ± 12.5	43.6 ± 12.0	43.6 ± 13.2	47.3 ± 16.3
LDL, mg/dl	125.4 ± 35.5	134.0 ± 35.0	122.0 ± 26.5	119.4 ± 36.5	98.8 ± 31.7*	97.1 ± 31.8	125.4 ± 19.6	121.5 ± 21.3	121.5 ± 21.0
IL-6, pg/ml	1.87 ± 2.11	2.08 ± 2.98	2.03 ± 2.37	1.83 ± 2.14	1.62 ± 1.47	1.30 ± 1.23	2.26 ± 1.79	2.38 ± 2.48	2.44 ± 1.34
IL-8, pg/ml	3.29 ± 1.11	3.30 ± 1.41	44.6 ± 116.1	10.4 ± 21.1	131.2 ± 358.2	4.22 ± 3.36	2.51 ± 1.28	3.46 ± 3.30	3.01 ± 1.94
CRP, mg/l	5.52 ± 6.13	4.50 ± 5.83	5.22 ± 5.73	4.46 ± 8.02	4.18 ± 8.02	1.76 ± 1.72	6.51 ± 6.19	5.64 ± 6.22	7.77 ± 6.77
APN, μg/l	7732 ± 4166	8021 ± 4796	8634 ± 4458	5846 ± 3762	5530 ± 4205	5575 ± 3215	6403 ± 4439	5975 ± 4490*	6532 ± 4586
FPG, mg/dl	83.1 ± 13.7	80.7 ± 5.22	79.0 ± 6.11	89.6 ± 13.4	85.0 ± 7.15	93.8 ± 18.1	89.8 ± 11.0	87.1 ± 9.80	89.6 ± 9.90
Insulin, μU/ml	7.19 ± 3.00	6.29 ± 3.47	6.80 ± 5.04	9.73 ± 6.87	10.1 ± 11.2	14.9 ± 27.8	11.3 ± 4.21	10.7 ± 6.49	12.0 ± 6.37
FMD, %	6.16 ± 5.30	6.94 ± 5.48	5.28 ± 4.92	5.63 ± 3.35	7.97 ± 3.83	6.76 ± 3.61	6.90 ± 4.22	8.67 ± 4.48	9.61 ± 6.75
CFU-As	51.6 ± 25.9	41.8 ± 30.0	61.0 ± 42.7	89.4 ± 42.2	60.4 ± 30. 6	44.3 ± 22.3*	78.1 ± 48.1	53.3 ± 36.1	55.4 ± 30.0
Cysteine, μmol/l	13.3 ± 3.53	11.0 ± 1.40	12.8 ± 2.06	12.1 ± 4.20	12.1 ± 3.35	9.93 ± 1.73	13.4 ± 3.18	13.3 ± 5.02	12.2 ± 2.80
Cystine, μmol/l	95.3 ± 18.9	103.6 ± 22.1	106.4 ± 30.6	95.1 ± 16.7	84.6 ± 18.3*	87.2 ± 11.0*	88.8 ± 18.2	91.5 ± 18.7	96.1 ± 18.5
Glutathione, μmol/l	1.98 ± 1.04	1.89 ± 0.48	1.87 ± 0.42	2.05 ± 1.24	2.09 ± 0.75	1.54 ± 0.52	2.38 ± 1.18	1.74 ± 0.59	1.89 ± 1.02

^a^Values are mean ± SD

^b^Means were calculated for all participants with non-missing data at the specified time point (baseline, 4 weeks, and 8 weeks)

*P < 0.05 for group-by-time interaction term where control and baseline are referent from mixed-effects models

Abbreviations: *APN* adiponectin, *FMD* flow-mediated vasodilation, *MUFA* monounsaturated fatty acids, *CFU-As* circulating pro-angiogenic cell activity, *TC* total cholesterol, *TG* triglycerides, *WC* waist circumference

### Effects of Mediterranean diet

Among participants who were assigned to the Mediterranean diet arm, there was a significant decrease from baseline to 8 weeks in body weight (− 2.2 [2.6] kg, *p* = 0.03), total cholesterol (− 24.9 [19.8] mg/dl, *p* = 0.003), LDL cholesterol (− 24.9 [17.3] mg/dl, p = 0.003), and CFU-As (− 40.3 [36.4] cfu, *p* = 0.01). There was also a non-significant increase in FMD from baseline to 4 weeks (2.3 [4.0] percent, *p* = 0.08). The average percent increase in FMD relative to baseline among participants assigned to the Mediterranean diet arm was 66.1% at 4 weeks and 44.3% at 8 weeks.

**Fig. 1:**
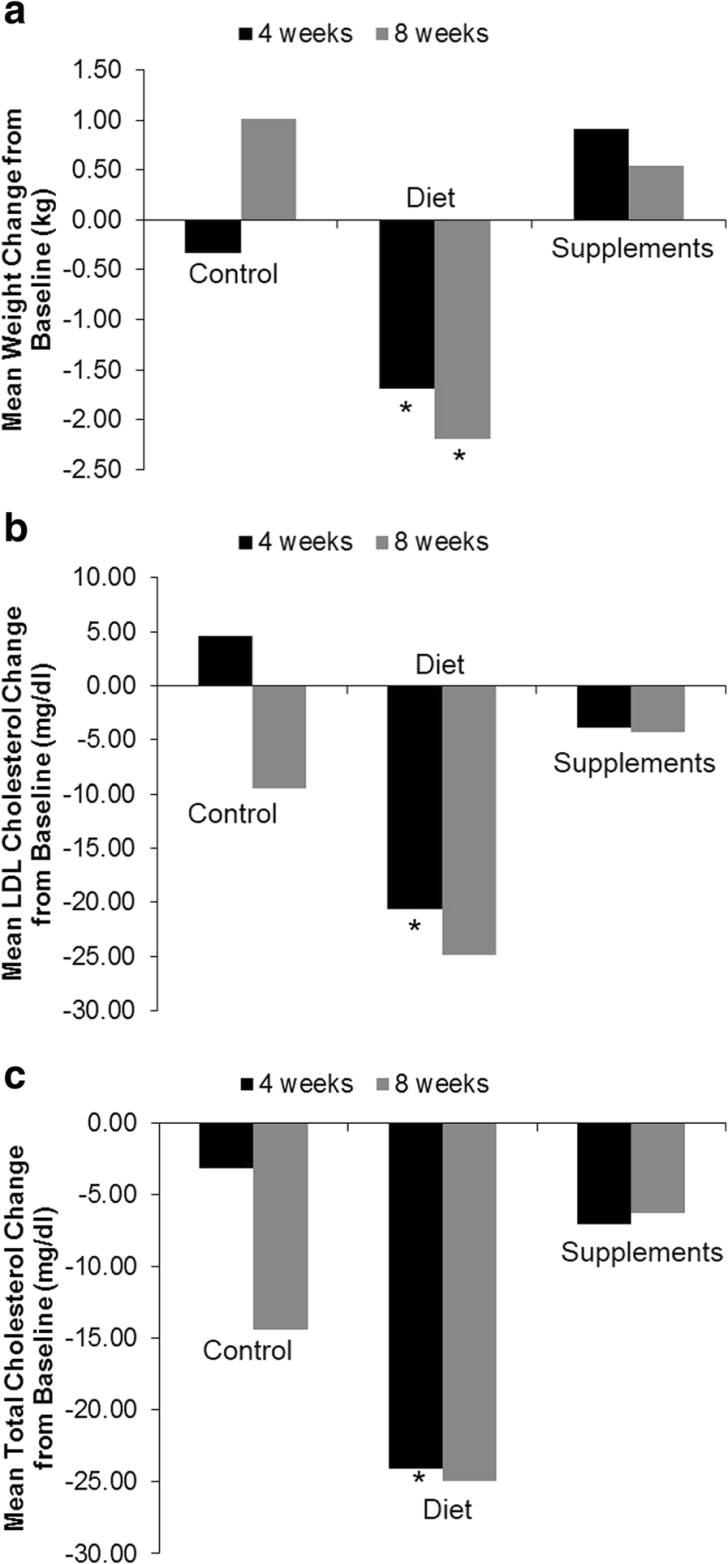
Mean change from baseline to 4 weeks (black bars) and 8 weeks (gray bars) in **a** body weight, **b** total cholesterol, and **c** LDL cholesterol. Mean change was calculated for all participants with non-missing data at both baseline and 4 weeks (black bars) or both baseline and 8 weeks (gray bars), thus may differ slightly from means presented in Table 1 (means calculated for all participants with non-missing data at a single time point). *Indicates *p* < 0.05 for group-by-time interaction term where control and baseline are referent from mixed-effects models

When compared to changes in controls, participants in the Mediterranean diet arm had significantly greater weight loss at both 4 and 8 weeks and greater decreases in total cholesterol and LDL cholesterol at 4 weeks (Table 1; Fig. 1). Moreover, compared to controls, participants in the Mediterranean diet arm had significantly greater decreases in CFU-As at 8 weeks, and in plasma cystine (indicative of decreased oxidative stress) at both 4 and 8 weeks. However, compared to changes in controls, participants in the Mediterranean diet arm had no significant changes in any of the inflammatory biomarkers or adipokines (IL-6, IL-8, CRP, and adiponectin), markers of hyperglycemia (FPG and insulin), or FMD. In a subgroup analysis of participants with FMD < 6% at baseline (*n* = 8 Mediterranean diet arm and *n* = 5 controls), there was a non-significant increase in FMD (*p* = 0.07) among those in the diet group compared to controls at 4 weeks.

### Effects of dietary supplementation with fish oil, walnuts, and grape juice

Among participants who received supplements, there was a significant decrease from baseline to 8 weeks in triglycerides (− 17.4 [16.8] mg/dl, *p* = 0.01), but in none of the other measures. When compared to changes in controls, the supplements resulted in significantly greater decreases in adiponectin levels at 4 weeks, but no significant changes in weight, waist circumference, lipid levels, inflammatory or oxidative stress biomarkers, markers of hyperglycemia, FMD, or CFU-As (Table 1; Fig. 1).

## Discussion

Eight weeks of substitution of an American-type diet with a Mediterranean diet was found to reduce CVD risk factors, specifically body weight and cholesterol levels, in a pilot randomized controlled trial in overweight or obese US adults. These changes with the Mediterranean diet were accompanied by significant improvement in systemic oxidative stress, measured as a decrease in cystine levels and a reduction in circulating pro-angiogenic cell activity. Supplementation of an American-type diet with key foods/nutrients thought to be responsible for the positive health effects (fish oil, walnuts, and grape juice) was not associated with significant changes in CVD risk factors, potentially due to the lack of weight loss in this group.

To our knowledge, this is one of the first randomized controlled trials to test the effects of a Mediterranean-type diet in a US sample population. A previous study conducted among 69 healthy, non-obese women in the United States found no significant effect on blood lipids, insulin, glucose, or CRP among those randomized to follow a modified Mediterranean diet using an exchange list compared to controls following their usual diet [7]. There are several potential explanations for the discrepancy between our findings and the findings of that study, including the fact that our study was conducted among overweight or obese adults and dietary interventions may be more effective among those at increased risk. In addition, we provided foods to participants for 4 weeks whereas the previous study provided only telephone counseling. However, given that we observed sustained effects on weight loss even after foods were no longer provided suggests that intensive counseling about how to follow a Mediterranean-type diet may be effective for CVD prevention among overweight or obese US adults.

We observed significant improvements in blood lipids, including total cholesterol and LDL cholesterol, among participants assigned to the Mediterranean diet arm compared to controls at 4 weeks. This is consistent with the observation that these participants had declines in saturated fat and cholesterol intake and increases in monounsaturated, omega-3, and omega-6 fatty acid intakes at 4 weeks. It is also consistent with several previous studies conducted in Europe and Brazil [5, 22–25]. Of note, the improvements in nutrient intake among Mediterranean diet arm participants were attenuated by 8 weeks, as participants were no longer provided prepared meals, but simply given counseling and reinforcement by the research nutritionist. This suggests that sustaining changes to an American-type diet that are consistent with a Mediterranean-type diet may require more intensive support.

The significant decrease in plasma cystine level and hence systemic oxidative stress in the Mediterranean diet arm suggests that this may be an important underlying mechanism explaining the protective effects of a Mediterranean diet on major cardiovascular events observed in the PREDIMED trial [1]. We have recently shown that higher levels of cystine are associated with mortality in patients with coronary artery disease [26]. Three secondary analyses of PREDIMED have focused on oxidative stress [27–29]. In a randomly selected subsample of 75 PREDIMED participants with metabolic syndrome, the plasma activity of superoxide dismutase and catalase was significantly increased and the activity of xanthine oxidase significantly decreased in both intervention groups (Mediterranean diet plus olive oil and Mediterranean diet plus nuts) compared to controls [29]. However, there were no significant differences across groups in plasma biomarkers of oxidative damage (nitrite levels, nitrotyrosine index, or carbonylated proteins) with the exception of nitrate levels, which were significantly higher in the two intervention groups compared to controls after 5 years of follow up [29]. The other two secondary analyses of the PREDIMED trial found significantly increased plasma total antioxidant capacity measured by colorimetric test in both intervention groups compared to controls after 3 years of follow up [27], and a non-significant (*p* = 0.059) decrease in F2-isoprostane levels among women with metabolic syndrome in the intervention groups compared to controls after 1 year of follow up [28]. Improved antioxidant capacity with a Mediterranean diet compared to control diet has also been reported in several other small trials in Chile (*n* = 42 healthy male students) [30] and Greece (*n* = 90 adults with abdominal obesity) [31].

Participants in the Mediterranean diet arm had significantly greater weight loss over the eight-week intervention period compared to controls despite having similar total caloric intakes (this was not designed to be a weight loss trial). This observation is consistent with a pre-specified secondary outcomes analysis of the PREDIMED trial that found a significant difference in weight loss in the Mediterranean diet with olive oil group compared to controls [32]. Together, these results lend further support to the hypothesis that it is not just diet quantity, but also diet quality, that matters for weight management. In contrast, in the supplement arm, a significant increase in caloric intake was observed at both 4 and 8 weeks. This may at least partially explain the lack of significant weight loss in this group.

We have previously shown that higher levels of circulating pro-angiogenic cell activity, measured as CFU-As, were associated with increased CVD risk [16, 17]. Most importantly, higher CFU-As were associated with an increased risk of cardiovascular events in patients with coronary artery disease [16, 17]. Thus, a higher number of CFU-As are potentially a reflection of a stimulated state of the individual’s endogenous stem cell-dependent reparative or regenerative system. Herein, we demonstrate that a Mediterranean diet intervention reduces the elevated CFU-A count, implying significant reduction of the stimulated state of the endogenous reparative system, and of reduced long-term risk of adverse cardiovascular outcomes.

This was a well-designed, carefully conducted study including thorough characterization of dietary intake and CVD risk. The lack of improvement in endothelial function and some of the biomarkers of inflammation could be due to the limited sample size, relatively short period of intervention, and the fact that some of these outcomes were not abnormal at baseline in a number of the subjects studied. For example, there was a trend towards improvement in endothelial function in the subset of participants with baseline abnormalities in FMD after the Mediterranean diet intervention. Findings need to be confirmed in a larger study.

## Conclusions

These results suggest that adopting a dietary pattern reflecting a Mediterranean diet improves weight and cardio-metabolic health and may be more beneficial than supplementing habitual American diets with fish oil, walnuts, and grape juice. Future research might focus on conducting larger trials of verbal and written counseling on the preparation and health benefits of a Mediterranean diet among a more diverse group of Americans, and also on the potential for supplementing with other key components of the Mediterranean diet (e.g., olive oil) on improving health in this population.

## Abbreviations

ANOVA: Analysis of variance
APN: Adiponectin
BMI: Body mass index
CFU-A: Circulating pro-angiogenic cell activity
CRC: Clinical research center
CRP: C-reactive protein
CVD: Cardiovascular disease
DHA: Docosahexaenoic acid
EPA: Eicosapentaenoic acid
FMD: Flow-mediated vasodilation
FPG: Fasting plasma glucose
LDL: Low-density lipoprotein
LOD: Limit of detection
MUFA: Monounsaturated fatty acids
TC: Total cholesterol
TG: Triglycerides
WC: Waist circumference
